# Hormonal Replacement Therapy in Menopausal Women with History of Endometriosis: A Review of Literature

**DOI:** 10.3390/medicina55080477

**Published:** 2019-08-14

**Authors:** Margherita Zanello, Giulia Borghese, Federica Manzara, Eugenia Degli Esposti, Elisa Moro, Diego Raimondo, Layla Omar Abdullahi, Alessandro Arena, Patrizia Terzano, Maria Cristina Meriggiola, Renato Seracchioli

**Affiliations:** Gynecology and Human Reproduction Physiopathology, Dipartimento di Scienze Mediche e Chirurgiche (DIMEC), S. Orsola Hospital, University of Bologna, Via Massarenti 13, 40138 Bologna, Italy

**Keywords:** menopause, hormone replacement therapy, menopause hormonal therapy, endometriosis, pelvic pain

## Abstract

Hormonal replacement therapy (HRT) is effective in treating the symptoms of menopause. Endometriosis is defined as the presence of functional endometrial tissue outside the uterine cavity with a tendency towards invasion and infiltration. Being an estrogen-dependent disease, it tends to regress after menopause. Nevertheless, it affects up to 2.2% of postmenopausal women. Conclusive data are not available in the literature on the appropriateness of HRT in women with endometriosis or a past history of the disease. The hypothesis that exogenous estrogen stimulation could reactivate endometriotic foci has been proposed. The aim of this state-of-the-art review was to revise the current literature about endometriosis in perimenopause and menopause and to investigate the possible role of HRT in this setting of patients. An electronic databases search (MEDLINE, Scopus, ClinicalTrials.gov, EMBASE, Sciencedirect, the Cochrane Library at the CENTRAL Register of Controlled Trials, Scielo) was performed, with the date range of from each database’s inception until May 2019. All of the studies evaluating the impact of different HRT regimens in patients with a history of endometriosis were selected. 45 articles were found: one Cochrane systematic review, one systematic review, five narrative reviews, two clinical trials, two retrospective cohort studies, 34 case reports and case series. Some authors reported an increased risk of malignant transformation of endometriomas after menopause in patients assuming HRT with unopposed estrogen. Low-quality evidence suggests that HRT can be prescribed to symptomatic women with a history of endometriosis, especially in young patients with premature menopause. Continuous or cyclic combined preparations or tibolone are the best choices. HRT improves quality of life in symptomatic post-menopausal women, who should not be denied the replacement therapy only due to their history of endometriosis. Based on low-grade literature evidence, we recommend to prescribe combined HRT schemes; tibolone could be considered.

## 1. Introduction

Endometriosis is defined as the presence of functional endometrial tissue (stroma and glands) outside the uterine cavity that has a tendency towards invasion and infiltration. This disease affects 10–15% of women of reproductive age, and its main symptoms are pelvic pain and infertility [1,2]. Being an estrogen-dependent disease, endometriosis tends to undergo regression after spontaneous or surgically-induced menopause [3,4]. In fact, menopause is associated with a fall in estrogen levels, which often relieves endometriosis-related symptoms, but can trigger menopausal ones, such as mood alterations, vaginal dryness, hot flushes, and night sweats. Nevertheless, endometriosis seems to affect 2.2% of postmenopausal women [5]. Postmenopausal endometriotic lesions include ectopic endometrial tissue islets remaining active after menopause and de-novo lesions diagnosed after menopause [6,7]. 

Many studies have investigated the relationship between menopause and endometriosis, but conclusive data on the appropriateness of the hormonal replacement therapy (HRT) in women with endometriosis or a past history of the disease are not available. In this setting of patients, the main issue is the hypothesis that exogenous estrogen stimulation could reactivate endometriotic foci and provoke a disease recurrence. Moreover, some authors have related the malignant transformation of the ectopic endometriotic tissue with the estrogenic stimulation [8].

The aim of this state-of-the-art review was to revise the current literature on endometriosis in perimenopause and menopause, and examine the possible role of HRT in menopausal patients with a history of endometriosis.

## 2. Materials and Methods

To identify relevant papers for inclusion in our literature review, a thorough search of electronic databases (i.e., MEDLINE, Scopus, ClinicalTrials.gov, EMBASE, Sciencedirect, the Cochrane Library at the CENTRAL Register of Controlled Trials, Scielo) was performed from their inception until May 2019. Search terms used were the following text words: “menopause,” “endometriosis”, “hormonal replacement therapy”, “menopause hormonal therapy”, “bilateral oophorectomy”. No restrictions for language or geographic location were applied. In addition, the reference lists of all articles identified were examined to identify studies not captured by electronic searches. The electronic search and the eligibility of the studies were independently assessed by two of the authors (G.B., F.M.). Final inclusion was decided after detailed examination of the studies. Differences were discussed, and consensus reached. We included all randomized clinical trials, retrospective studies, literature reviews, case reports and series dealing with the use of hormonal replacement therapy in menopause in patients with history of endometriosis.

## 3. Results

Based on the aforementioned criteria, 45 articles were found. Only one Cochrane systematic review and one systematic review of the literature were retrieved. Five narrative reviews were available in the literature. We found two clinical trials and two retrospective cohort studies. The remaining articles were case reports and cases series of menopausal patients with endometriosis recurrence after HRT. 

### 3.1. Estrogenic Stimulation after Menopause

Literature on the pathophysiologic mechanisms leading to postmenopausal endometriosis is scarce. While during reproductive years estrogenic production is mainly due to ovarian secretion, different studies have investigated the possible sources of estrogen after menopause. Adipose tissue and adrenal glands seemed involved, as well as exogenous sources, such as phytoestrogens, HRT and tamoxifen [9]. A role for exogenous estrogens in re-activating endometriotic foci leading to a recurrence of symptoms has been proposed. Bulum et al. described an estrogenic production by the endometriotic implants themselves thorough the aromatase enzyme [10], not confirmed by other authors [11]. Scemama et al. proposed that post-menopausal endometriosis might consist in either a persistence or a recurrence of a pre-existing condition [12].

### 3.2. Risk of Malignant Transformation

Endometriosis is a chronic, recurrent, benign disease defined as the presence of endometrial tissue outside the uterine cavity, with the tendency to proliferate and invade surrounding tissues. However, endometriotic lesions, especially ovarian implants, can turn into malignant lesion in about 1% of cases [13]. Endometriosis-associated ovarian cancer typically belongs to type I ovarian cancers with favorable characteristics: low-grade disease, early-stage disease at onset and endometrioid or clear cell histology [14]. In a meta-analysis of 13 case-control studies, the odds ratio for developing an ovarian cancer in patients with a history of endometriosis was of 3.05 (confidence interval, CI 2.43–3.84) for clear cell carcinoma, 2.04 for endometrioid carcinoma (CI 1.67–2.48) and 2.11 for low-grade serous carcinoma (CI 1.39–3.20) [15]. Zanetta et al. reported an increased risk of malignant transformation of the endometriomas after menopause, especially in patients assuming HRT with unopposed estrogens [16]. 

### 3.3. Therapy for Endometriosis after Menopause

#### 3.3.1. Surgery

The first line treatment option for post-menopausal symptomatic endometriosis should be surgical exploration, due to the uncertainty of diagnosis and the risk of potential malignant transformation. The surgical approach, more commonly referred to as laparoscopy, should be performed in order to provide histological confirmation of the condition and relief of painful symptoms [17].

#### 3.3.2. Medical Therapy

A medical approach should be reserved to cases of recurrence of disease after surgery, when surgery is contraindicated. Progestogens administration is the most common medical strategy. Bulun et al. proposed the use of aromatase inhibitors (AI), that are expected to block the extra ovarian estrogenic secretion, thus decreasing lesion size and pain [10].

##### Progestogens

In post-menopausal patients with recurrence or new onset of endometriosis with comorbidities contraindicating the surgical approach, progestogens are a reliable strategy, thanks to their direct action on the progesterone receptor of endometrial ectopic tissue. Several oral progestogens, such as gestodene and the levonorgestrel intrauterine system (LNG-IUS), have been proposed. Moreover, LNG-IUS has been proposed in conjunction with a systemic estrogen for HRT [18]. No extensive data are available about progestogens use in this specific setting of patients, and further studies are needed. 

##### Aromatase Inhibitors 

The rationale for use of the AI is their action on extra-ovarian estrogen production [19]. According to Bulun and colleagues, also intralesional estrogen production could be blocked by AI administration [10]. Six case reports of AI administration in post-menopausal women with a history of endometriosis are published; five are described by Polyzos et al. in a literature review [20,21]. All of the patients had recurrent symptomatic endometriosis; two were taking HRT. AI treatment was effective in pain relief and in some cases in reducing the size of lesions. Conversely, AI could imply menopausal-like adverse effects such as hot flushes, vaginal dryness, and decreased bone mineral density because of the suppression of extra-ovarian estrogen secretion [17]. Low-dose estrogen add-back therapy could be an option.

### 3.4. HRT in Patients with History of Endometriosis 

Menopausal symptoms, including hot flushes, sleep and mood disorders, and painful sexual intercourse, significantly affect the quality of life of millions of women worldwide [22]. Moreover, the hypo-estrogenic state was demonstrated to be a risk factor for cardiovascular and bone disease [23,24]. HRT was proven to improve the quality of life and to be safe in symptomatic postmenopausal women [25,26]. 

The risks and benefits of HRT in coronary heart disease (CHD) prevention are controversial, mainly related to age menopause [27]. On the other hand, HRT is considered effective for the prevention of postmenopausal osteoporosis, but it is generally recommended only for women at significant risk [28]. 

Concerning postmenopausal symptomatic women with a history of endometriosis, when deciding whether or not to prescribe HRT other factors need to be considered, such as the presence of residual disease and the painful symptoms. In fact, in this setting, the main issue is the hypothesis that exogenous estrogen stimulation could reactivate endometriotic foci; secondly, a recent systematic review of the literature suggested that the malignant transformation of the ectopic endometriotic tissue is related to estrogenic stimulation [8]. Few authors have investigated the role of HRT in patients with a history of endometriosis, and the only evidence comes from women submitted to hysterectomy and bilateral salpingo-oophorectomy (BSO) for symptomatic endometriosis, for whom HRT is often prescribed. In a 2009 a Cochrane review found only two randomized clinical trials, and concluded that HRT may increase the risk of symptomatic recurrence after surgically-induced menopause [29]. The authors suggested that if a residual of disease is present after surgery, increased risk exists, and HRT should be avoided. A recent systematic review [8] found only one randomized clinical trial and two cohort studies comparing post-menopausal women assuming HRT versus not assuming the therapy (Table 1). Matorras et al. in a single-center randomized clinical trial (RCT) including 172 women with a history of endometriosis submitted to BSO compared patients randomly assigned to treatment with combined HRT (50 μg estradiol daily and oral micronized progesterone for 14 days out of every 30 days) or no treatment. All of the endometriosis recurrences were reported in the HRT arm (3.5%), without detecting a statistically significant difference between the two groups in terms of recurrence rate. The authors proposed the presence of residual endometrial tissue as a risk factor for disease recurrence [30]. Acien et al. described 19 patients who had undergone hysterectomy and BSO for symptomatic endometriosis. Of them, 11 were subsequently treated with HRT (1–2 years of combined HRT, followed by low dose estrogen-only HRT or tibolone), while eight did not undergo hormonal therapy. No patients in either group were diagnosed with endometriosis recurrence [31]. Another retrospective cohort included 107 women who had undergone hysterectomy and BSO for endometriosis. 90 patients received HRT (various schemes including unopposed estrogens or combined HRT) while 17 were not treated. Recurrence was identified in four women assuming unopposed estrogen therapy [32]. 

Current data are therefore insufficient to support any conclusion about the administration of HRT in women with a history of endometriosis; patients should be evaluated in detail before initiating a “tailored” HRT. However, the evidence is not enough to justify avoiding HRT for symptomatic women. In young menopausal women with premature or surgically-induced menopause the benefits of HRT probably overcome the risks and HRT should be offered until the average age of natural menopause [33,34]. 

In women with residual endometriosis after surgery, the use of HRT should be discussed and the risk of recurrence should be carefully considered before starting an estrogen-based replacement therapy. 

The only study addressing the timing of HRT investigated patients with surgical menopause [35] and found that patients who began HRT immediately after BSO had no greater risk of endometriosis recurrence than those who delayed HRT. Thus, the authors suggested that the replacement therapy could be started immediately after the surgical menopause onset. 

#### Which HRT is More Suitable in Patients with History of Endometriosis?

The choice of the most suitable HRT scheme for menopausal women with a history of endometriosis is a relevant issue. Unopposed estrogen, combined HRT (estrogens plus progestogens) and tibolone (which typically has an estrogenic effect on climacteric symptoms and bone, yet a progestogenic effect on tissues) have been investigated, but high quality data are missing from the literature. We found only one RCT comparing different HRT preparations [36]; the authors evaluated 21 women with residual pelvic endometriosis after BSO with or without hysterectomy, randomly allocated to receive transdermal estradiol 50 μg twice weekly (plus cyclic medroxyprogesterone acetate (10 mg per day) for 12 days per month in women with a conserved uterus) or continuous tibolone (2.5 mg/day). After 12 months, four patients (40%) in the estradiol group, compared to only one (9%) in the tibolone group, referred moderate pelvic pain. This difference was not proven to be statistically significant. Moreover, one patient in the HRT group discontinued treatment because of the onset of post-coital bleeding and dyspareunia due to a vaginal nodule. Gemmell et al. found out that the majority of cases of endometriosis recurrence took place in women with prior hysterectomy, who took unopposed estrogen [8]. In previous studies, tibolone was associated with an increased risk of endometriosis recurrence [37]. In the previously-mentioned RCT [36], Fedele et al. suggested that tibolone could be a safer alternative to traditional HRT in patients with residual endometriotic disease. Unopposed estrogen seems therefore to carry a higher risk of recurrence than combined preparations. Although without high quality literature findings, continuous or cyclic combined preparations or tibolone would appear better choices [38]. 

In women unable to tolerate oral progestogens, the use of a low dose LNG-IUS in conjunction with a systemic estrogen could be an option [18]. New HRT combinations, composed of estrogens and Dienogest have been proposed; evidence in the literature is still scarce, but both this strategy and LNG-IUS plus oral estrogens could be reliable options with a double role in controlling menopausal symptoms and prevent endometriosis recurrence [39]. 

The use of oral isoflavone as a dietetic supplement was associated with the reduced risk of endometriosis recurrence [40]. Conversely, in a case report Noel et al. suggested a relationship between use of oral isoflavones and endometriosis relapse [41]. Further studies are needed to investigate the possible association between phytoestrogens and endometriosis and a possible role of selective estrogen receptor modulators (SERMs) in symptomatic post-menopausal women with a history of endometriosis.

### 3.5. Follow-up 

A regular follow up is recommended, even if literature data about the timing of follow up in patients assuming HRT with a previous history of endometriosis are lacking. Every follow up visit should investigate the eventual recurrence of symptoms (chronic pelvic pain, dyschezia, dysuria, dyspareunia), especially in women with residual disease [42,43]. If a recurrence is ascertained, HRT should be stopped and the pain investigated [44]. 

## 4. Conclusions 

It has been established that HRT plays a role in improving bone healthy and quality of life in symptomatic post-menopausal women and in reducing the cardiovascular risk in younger and early menopausal patients. Endometriosis is a condition affecting mainly women in their fertile years, but not exclusively; in fact, Punnonen et al. reported a prevalence of up to 2.2% in post-menopausal years [5]. The management of this subset of patients poses a challenge between the treatment of the menopausal symptoms and the risk of endometriosis recurrence. Some evidence suggests that women should not be denied the replacement therapy only due to a history of endometriosis. The decision whether or not to prescribe HRT requires careful counselling about benefits and risks, and the patient’s involvement in the therapeutic decision. This is pivotal, particularly in patients with early natural or surgical menopause. The HRT regimen should be discussed with the patients. Based on low-grade evidence in the literature, we recommend prescribing combined HRT instead of unopposed estrogen. Tibolone could be considered. Further studies are needed to investigate the possible role of phytoestrogens and SERMs in symptomatic post-menopausal women with a history of endometriosis. 

## Figures and Tables

**Table 1:** Studies comparing post-menopausal women with history of endometriosis assuming versus not assuming hormonal replacement therapy (HRT).

Author	Study Design	Patients	Type of HRT	Duration of HRT (Months)	Recurrence Rate (%)	*p*-Value
*Matorras et al.* (2002) [28]	Randomized clinical trial	172 submitted to BSO (115 study group, 57 controls)	Study group: 50 μg estradiol daily plus oral micronized progesterone for 14 days out of 30 days	ns	Study group: 4/115 (3.5%)	*p* = 0.32
			Control group: no treatment		Controls: 0/57 (0.0%)	
*Acièn et al.* (2013) [29]	Retrospective observational study	27 submitted to HBSO (14 study group, 13 controls)	Study group: combined HRT, followed by low dose ERT or tibolone	67.2 ± 43.2	Study group: 0/14 (0.0%)	na
			Control group: no treatment		Controls: 0/13 (0.0%)	
*Rattanachaiyanont et al.* (2003) [30]	Retrospective observational study	107 submitted to HBSO (90 study group, 17 controls)	Study group 1: ERT (*n* = 50)	41.2	Study group 1: 4/50 (8.0%) (1/50 (2.0%) endometriosis recurrence, 3/50 (6.0%) symptoms recurrence)	na
			Study group 2: cyclic E/P (*n* = 16)		Study group 2: 0/16 (0.0%)	
			Study group 3: ccE/P (*n* = 24)		Study group 3: 0/24 (0.0%)	
			Control group: no HRT, (*n* = 17)		Control group: 0/17 (0.0%)	

Legend: BSO = bilateral salpingo-oophorectomy; ERT = estrogen-only replacement therapy; HBSO = hysterectomy and bilateral salpingo-oophorectomy; HRT = hormonal replacement therapy; cyclic E/P = cyclic combined estrogen/progestogen regimen; cc E/P = continuous combined estrogen/progestogen; na = not assessed.
